# Prevalence, Molecular Characterization, and Antibiotic Susceptibility of *Vibrio parahaemolyticus* from Ready-to-Eat Foods in China

**DOI:** 10.3389/fmicb.2016.00549

**Published:** 2016-04-21

**Authors:** Tengfei Xie, Xiaoke Xu, Qingping Wu, Jumei Zhang, Jianheng Cheng

**Affiliations:** ^1^School of Bioscience and Bioengineering, South China University of TechnologyGuangzhou, China; ^2^Guangdong Institute of Microbiology, State Key Laboratory of Applied Microbiology Southern China: Guangdong Provincial Key Laboratory of Microbial Culture Collection and Application, Guangdong Open Laboratory of Applied MicrobiologyGuangzhou, China

**Keywords:** *Vibrio parahaemolyticus*, ready-to-eat foods, antibiotic resistance, ERIC-PCR, MLST, serotype, virulence gene

## Abstract

*Vibrio parahaemolyticus* is the leading cause of foodborne outbreaks, particularly outbreaks associated with consumption of fish and shellfish, and represents a major threat to human health worldwide. This bacterium harbors two main virulence factors: the thermostable direct hemolysin (TDH) and TDH-related hemolysin (TRH). Additionally, various serotypes have been identified. The extensive use of antibiotics is a contributing factor to the increasing incidence of antimicrobial-resistant *V. parahaemolyticus*. In the current study, we aimed to determine the incidence and features of *V. parahaemolyticus* in ready-to-eat (RTE) foods in China. We found 39 *V. parahaemolyticus* strains on Chinese RTE foods through investigation of 511 RTE foods samples from 24 cities in China. All isolates were analyzed for the presence of *tdh* and *trh* gene by PCR, serotyping was performed using multiplex PCR, antibiotic susceptibility analysis was carried out using the disk diffusion method, and molecular typing was performed using enterobacterial repetitive intergenic consensus sequence PCR (ERIC-PCR) typing and multilocus sequence typing (MLST). The results showed that none of the isolates were positive for *tdh* and *trh*. Most of the isolates (33.3%) were serotype O2. Antimicrobial susceptibility results indicated that most strains were resistant to streptomycin (89.7%), cefazolin (51.3%), and ampicillin (51.3%). The isolates were grouped into five clusters by ERIC-PCR and four clusters by MLST. We updated 10 novel loci and 33 sequence types (STs) in the MLST database. Thus, our findings demonstrated the presence of *V. parahaemolyticus* in Chinese RTE foods, provided insights into the dissemination of antibiotic-resistant strains, and improved our knowledge of methods of microbiological risk assessment in RTE foods.

## Introduction

*Vibrio parahaemolyticus* is a gram-negative halophilic bacterium that naturally occurs worldwide in estuarine environments. This microorganism is recognized as one of the leading causes of foodborne illness worldwide and has been shown to cause acute gastroenteritis in humans. Complications such as septicemia can sometimes lead to death in patients with *V. parahaemolyticus* infection (Qadri et al., 2005; Lopatek et al., 2015). Previous studies have focused on the prevalence of *V. parahaemolyticus* in shellfish, oysters, water, seafood, and shrimp (Khouadja et al., 2013; Lopatek et al., 2015). However, no reports have identified isolates of *V. parahaemolyticus* on ready-to-eat (RTE) foods such as cooked meat, roasted poultry, and cold vegetable dishes in sauce, that are highly popular in China. Various foodborne pathogens may be present in RTE foods and may cause illnesses in consumers because RTE foods do not require heat treatment or other forms of curing before eating (Shi et al., 2015). Moreover, the high genetic diversity on RTE foods can facilitate identification of strain relatedness and epidemiological investigations.

Traditionally, *V. parahaemolyticus* is considered susceptible to antimicrobials. However, during the past few decades, antimicrobial resistance has emerged and evolved in many bacterial genera owing the excessive use of antimicrobials in human, agriculture, and aquaculture systems (Cabello, 2006; Kang et al., 2016). For example, tetracyclines are recommended as antibiotics in the treatment of severe *Vibrio* infections, and third-generation cephalosporins with doxycycline or fluoroquinolone alone are also used on occasion. Moreover, antibiotics are commonly used to treat fish (Han et al., 2007; Devi et al., 2009). Some *V. parahaemolyticus* isolates from seafood and other environments are commonly resistant to ampicillin, aminoglycosides (streptomycin and gentamicin), ciprofloxacin, chloramphenicol, and other antibiotics (Oh et al., 2011; Raissy et al., 2012; Shaw et al., 2014; Lopatek et al., 2015). Therefore, the potential presence of antibiotic-resistant *V. parahaemolyticus* on RTE foods may be an important public health problem related to disease management and control.

The virulence of *V. parahaemolyticus* is mainly attributed to the production of two major factors: thermo-stable direct hemolysin (TDH) encoded by the *tdh* gene and TDH-related hemolysin encoded by the *trh* gene (Honda and Iida, 1993). Clinical strains commonly contain either these genes, and the presence of these genes is associated with the pathogenicity of the strain in humans (Su and Liu, 2007; Jones et al., 2012; Pazhani et al., 2014). Detection of *V. parahaemolyticus* virulence factors is typically based on molecular biological analysis and amplification of *V. parahaemolyticus*-specific sequences (Shirai et al., 1990; West et al., 2013). To date, there are 13 O-serogroups and over 70 K-serogroups, differentiated on the basis of somatic (O) and capsular (K) antigens in *V. parahaemolyticus* (Ishibashi et al., 2000; Jones et al., 2012). The emergence of the first pandemic strain belonging to serovar O3:K6 (Okuda et al., 1997) supported the view that the serotype of *V. parahaemolyticus* is correlated with virulence. A multiplex polymerase chain reaction (PCR)-based O-antigen serotyping method has been adopted because other antiserum-based approaches are time consuming, expensive and can be associated with the risk of cross-reactions (Chen M. et al., 2012; Xu et al., 2014).

Molecular typing of *V. parahaemolyticus*, including pulsed field gel electrophoresis (PFGE) (Marshall et al., 1999), repetitive extragenic palindromic sequence PCR (REP-PCR) (Wong and Lin, 2001), and ribotyping (Bag et al., 1999), has been shown to be a useful tool for providing genetic relatedness information (Olive and Bean, 1999). Enterobacterial repetitive intergenic consensus sequence PCR (ERIC-PCR) has been proven useful for subtyping of *V. parahaemolyticus* strains with highly conserved repetitive intergenic consensus sequences (De Bruijn, 1992; Chen W. et al., 2012). Additionally, multilocus sequence typing (MLST), which is based on sequence analysis of selected housekeeping genes (e.g., *recA, gyrB, dnaE, dtdS, pntA, pyrC*, and *tnaA*), is becoming an important method for investigation of the evolution and epidemiology of *V. parahaemolyticus* owing to its high repeatability (González-Escalona et al., 2008; Banerjee et al., 2014).

*V. parahaemolyticus* is not frequently found in RTE foods, but is still considered hazardous in humans. Therefore, the objectives of the current study were as follows: (i) to determine the prevalence and contamination level of *V. parahaemolyticus* in RTE foods in China and (ii) to determine the genetic variation and phenotypic characteristics of *V. parahaemolyticus* isolates from RTE foods. The information generated in this study will provide insights into the distribution and population of *V. parahaemolyticus* across Chinese RTE foods and differentiation of *V. parahaemolyticus* strains on different RTE foods.

## Materials and methods

### Sample collection

**Figure 1:**
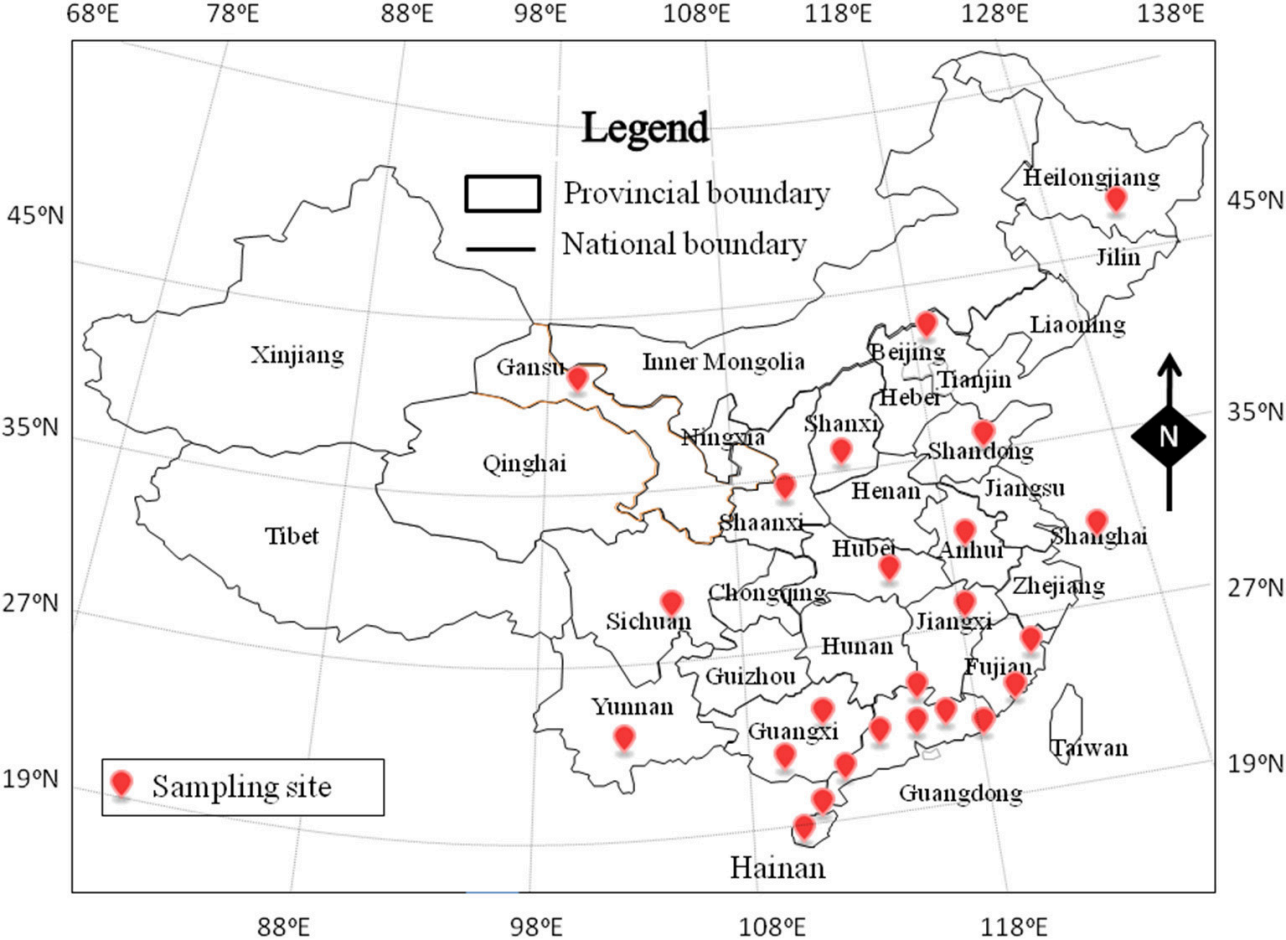
**The sampling site of ready-to-eat (RTE) foods in China**.

From November 2011 to May 2014, a total of 511 RTE food samples were collected from retail markets in 24 cities, covering most provincial capitals of China (Figure 1). The samples consisted of 371 deli meat samples, 97 cold vegetable dishes or noodles in sauce, and 43 fried rice or noodle samples. The samples were placed in sterile sealed plastic bags, transported to the laboratory in a cold box below 4°C, and analyzed immediately.

### Qualitative detection

The bacteriological media used herein, unless indicated, were purchased from Guangdong Huankai Co. Ltd. (Guangzhou, China). For qualitative detection, the *V. parahaemolyticus* was isolated according to GB 4789.7-2013 for food microbiological examination of *V. parahaemolyticus* (National Food Safety Standards of China) with minor modifications. In brief, 25 g of each sample was homogenized for 60 s in a stomacher bag (Huankai Co. Ltd., Guangzhou, China) with 225 mL of alkaline peptone water (APW) containing 3% NaCl. Homogenates were incubated at 37°C for 16−18 h. After incubation, a loopful from the top 1 cm was streaked onto thiosulfate-citrate-bile salts-sucrose (TCBS) agar plates and incubated at 37°C for 18−24 h. Presumptive colonies (green or blue green colonies, 2−3 mm in diameter) were streaked onto Chromogenic Vibrio Medium and incubated at 37°C for 24 h. The mauve colonies were selected for further *V. parahaemolyticus* identification by analysis of oxidase activity, Gram staining, 3.5% NaCl triple sugar iron (TSI) tests, halophilism tests, and API 20E diagnostic strips (Biomerieux Company, France).

### MPN enumeration (MPN) of *V. parahaemolyticus*

Enumeration of *V. parahaemolyticus* in RTE food samples was performed using the MPN method according to the Bacteriological Analytical Manual standard method (Kaysner and Depaola, 2004) and a previous study (Xu et al., 2014).

### Detection of tdh and *trh* genes

Detection of the *V. parahaemolyticus tdh* and *trh* genes was performed by PCR as described previously (Gutierrez West et al., 2013). The oligonucleotide primers were synthesized by Sangon Biotech (Shanghai, China) (Tdh-F: CTGTCCCTTTTCCTGCCCCCG, Tdh-R: AGC CAGACACCGCTGCCATTG; Trh-F: ACCTTTTCCTTCTCCWGGKTCSG, Trh-F: CCGCTC TCATATGCYTCGACAKT). Each reaction mixture included the following (total volume, 25 μL): 2 × PCR Mix (Qiagen), 12.5 μL; 0.5 μM each primer, dd H_2_O, 9.5 μL; and DNA template, 1 μL. Both genes were amplified using the following thermal-cycling program: denaturation at 95°C for 5 min; 40 cycles of 95°C for 1 min, 62°C for 1 min, and 72°C for 1 min; and a final extension of 72°C for 2 min. PCR was conducted in a Bio-Rad PTC-200 Thermal Cycler (Bio-Rad, Hercules, CA, USA). The amplified products were then analyzed electrophoretically on a 2% agarose gel containing Gold View. The images were captured digitally and analyzed using a Gel Image system (Bio-Rad). *V. parahaemolyticus* strains ATCC 33847 (*tdh*+) and ATCC 17802 (*trh*+) were used as positive controls, and distilled water was used as the negative control.

### Multiplex serotyping PCR

The serotypes of *V. parahaemolyticus* isolates were identified using the PCR-based O-antigen serotyping technique. The primer concentrations and amplification conditions used were as described previously (Chen M. et al., 2012). The primers used for this assay were synthesized by Sangon Biotech (Shanghai, China). The 12 primer pairs were divided into two groups to amplify target DNA; PCR group 1 was used to detect serogroups O1, O2, O4, O5, O6, and O10, whereas PCR group 2 was used to detect serogroups O3/O13, O7, O8, O9, O11, and O12. The PCR was performed in a 25-μL reaction mixture containing the following: 2 × PCR mix (Dongshen, Guangzhou, China), 12.5 μL; 0.5 μM each primer, dd H_2_O, 9.5 μL; and DNA template, 1 μL. All amplifications were carried out with the following protocol: 30 cycles of 95°C for 30 s, 60°C for 45 s, and 72°C for 1 min. The thermal cycler was prewarmed to 80°C before all the reaction tubes were added in order to reduce nonspecific amplification. PCR was conducted in a Bio-Rad PTC-200 Thermal Cycler (Bio-Rad, California, USA). The amplified products were then analyzed electrophoretically on a 2% agarose gel containing Gold View. The images were captured digitally and analyzed using the Gel Image system (Bio-Rad, California, USA). *V. parahaemolyticu*s ATCC 17802 and ATCC 33847 were used as control strains.

### Antimicrobial susceptibility

The susceptibility of the *V. parahaemolyticus* isolates to antibiotics was examined by the disk diffusion method according to the guidelines of the Clinical and Laboratory Standards Institute (CLSI, 2012) and a previous study (Xie et al., 2015). Briefly, Muller-Hinton agar and a panel of 12 antibiotics disks were selected for resistance tests. These 12 antibiotic disks (Oxoid, Hampshire, UK) were ampicillin (10 μg), azithromycin (15 μg), cefazolin (30 μg), cephalothin (30 μg), chloramphenicol (30 μg), ciprofloxacin (5 μg), gentamicin (10 μg), kanamycin (30 μg), nalidixic acid (30 μg), streptomycin (10 μg), trimethoprim-sulfamethoxazole (25 μg), and tetracycline (30 μg). The results were expressed as sensitive (S), intermediate (I), or resistant (R) following the methods of the CLSI. *Escherichia coli* ATCC 25922 and *V. parahaemolyticu*s ATCC 17802 were used as quality control organisms.

### ERIC-PCR analysis

ERIC-PCR analysis was performed on the *V. parahaemolyticus* isolates using a previously described protocol with some modifications (Chen W. et al., 2012; Xie et al., 2015). The reaction mixture (25 μL per reaction) consisted of 12.5 μL 2 × Long Taq Mix (Dongsheng Biotech, Guangzhou, China), 0.6 μM of each primer (5′-ATGTAAGCTCCTGGGGATTCAC-3′ and 5′-AAGTAAGTGACTGGGGTGAGCG-3′), and 100 ng template DNA. PCR was performed in a DNA thermocycler (Applied Biosystems, CA, USA) using the following procedure: one cycle of denaturation at 95°C for 5 min; 35 cycles of 94°C for 45 s, 52°C for 1 min, and 72°C for 3 min; and a final extension at 72°C for 10 min. The PCR products were separated by electrophoresis on 2.0% agarose gels followed by Goldview staining (0.005%, v/v; SBS Genetech, Beijing) and imaging with a UV Imaging System (GE Healthcare, WI, USA). The images were captured in TIFF file format for further analysis.

The size of each band in the ERIC patterns was determined, and the data were coded as 0 (absence) or 1 (presence). Cluster analysis was performed with NTSYS-pc (Version 2.10), a numerical taxonomy and multivariate analysis software package (Rohlf, 2000), based on Dice's similarity coefficient (SD), with a 1% position tolerance and the unweighted pair group method using arithmetic averages (UPGMA).

### MLST

MLST analysis was conducted via the *V. parahaemolyticus* MLST website and database (http://pubmlst.org/vparahaemolyticus/) (Jolley et al., 2004). PCR conditions were denaturation at 96°C for 1 min; primer (Table S1; synthesized by Sangon Biotech, Shanghai, China) annealing at 58°C for 1 min; and extension at 72°C for 1 min, for 30 cycles; with a final extension step at 72°C for 10 min. PCR was performed with a Bio- Rad PTC-200 Thermal Cycler (Bio-Rad, California, USA) according to the manufacturer's directions. PCR products were sequenced on a BGI instrument (Shenzhen, China). The alignments of these sequences were determined using BioEdit. The sequences were analyzed online (http://pubmlst.org/vparahaemolyticus/) to assign allele numbers and define STs. New sequences for alleles and new ST profiles were submitted to the *V. parahaemolyticus* MLST database.

The evolution tree of the concatenated sequences of the seven loci was built based on the method of the Kimura-2-parameter in Mega 6.0 (Tamura et al., 2013). The ratio between the number of synonymous and nonsynonymous substitutions, showing the type of selection at each locus, was calculated using the method of Nei and Gojobori in Mega 6. The hypotheses of neutrality (dS = dN), purifying selection (dS/dN >1), and positive selection (dS/dN < 1) were tested using DNAsp 5.10 (Lüdeke et al., 2015).

## Results

### *V. parahaemolyticus* in RTE food samples

Of the 511 samples tested, eight were positive by both qualitative and MPN methods; 12 showed positive results by the qualitative method only, while 10 were positive with the MPN method only. Thirty (5.9%) samples positive for *V. parahaemolyticus* were detected among 511 samples after qualitative and MPN analyses, including 22 (5.9%) of the 371 deli meat samples, seven (7.2%) of the 97 samples of cold vegetable dishes or noodles in sauce, and one (2.3%) of the 43 fried rice or noodle samples. In 18 positive samples detected by the MPN method, *V. parahaemolyticus* densities ranged between 3 and 100 MPN/g in all of the samples. The densities of rest positive samples were below 3.0 MPN/g. According to the National Food Safety Standards of China (GB 2727-2005, GB2726-2005), the pathogenic bacteria should not be detected in deli meat such as cooked meat or roasted meat.

### Detection of *tdh* and *trh* genes in *V. parahaemolyticus* isolates

A total of 39 *V. parahaemolyticus* isolates were confirmed and tested for the presence of the *trh* and *tdh* genes. None of the isolates possessed the *tdh* and *trh* genes.

### Serotyping by multiplex PCR

With the exception of serotypes O9, O10, and O11, all other serotypes were detected among the isolates. Serotype O2 was the most prevalent (13 isolates), followed by serotype O4 (eight isolates). The results of the O-antigen serotyping for all 39 isolates are shown in Table 1. The serotypes of *V. parahaemolyticu*s ATCC 17802 and ATCC 33847 were O1 and O4, respectively (Figure S2).

**Table 1:** **Results of the PCR-based O-antigen serotyping of 39 ***V. parahaemolyticus*** isolates**.

**Serogroup**		**Product size (bp)**	**No. of isolates**
Group 1	O1	474	3
	O2	238	13
	O4	671	8
	O5	852	3
	O6	1409	1
Group 2	O3/O13	868	1
	O7	385	1
	O8	680	2
	O12	256	7
Total			39

### Antimicrobial susceptibility

The susceptible, intermediate, and resistance rates of the 39 examined *V. parahaemolyticus* isolates with respect to 12 antibiotics are shown in Table 2. The resistance to streptomycin (S), ampicillin (AMP), cefazolin (KZ), cephalothin (KF), kanamycin (K), gentamicin (CN), and trimethoprim-sulfamethoxazole (SXT) reached 89.7, 51.3, 51.3, 41.0, 41.0, 2.6, and 2.6%, respectively. None of the strains showed resistance to azitromycin (AZM), chloramphenicol (C), ciprofloxacin (CIP), nalidixic acid (NA), or tetracycline (TE). Most of the isolates were resistant to streptomycin, with resistance and intermediate rates of 89.3 and 10.3%, respectively, which was consistent with a previous study (Xie et al., 2015). The next-highest susceptible rates were observed for trimethoprim-sulfamethoxazole (87.1%) and gentamicin (51.3%). In addition, there were four multidrug-resistant isolates showing resistance to five antibiotics, of which one isolate was collected from deli meat in Xiamen, two isolates were obtained from deli meat in Chengdu, and one isolate was obtained from deli meat in Jinan (Table S2).

**Table 2:** **Antimicrobial resistance profiles of 39 ***Vibrio parahaemolyticus*** isolates**.

**Antimicrobial agent**	***Vibrio parahaemolyticus*** **(*n* = 39)**		
	**NO.(%) of R**	**NO.(%) of I**	**NO.(%) of S**
Ampicillin (AMP)	20 (51.3)	14 (35.9)	5 (12.8)
Azitromycin (AZM)	0 (0.0)	14 (35.9)	25 (64.1)
Cefazolin (KZ)	20 (51.3)	18 (46.1)	1 (2.6)
Cephalothin (KF)	16 (41.0)	22 (56.4)	1 (2.6)
Chloramphenicol (C)	0 (0.0)	1 (2.6)	38 (97.4)
Ciprofloxacin (CIP)	0 (0.0)	13 (33.3)	26 (66.7)
Gentamicin (CN)	1 (2.6)	18 (46.1)	20 (51.3)
Kanamycin (K)	16 (41.0)	20 (51.3)	3 (7.7)
Nalidixic acid (NA)	0 (0.0)	2 (5.1)	37 (94.9)
Streptomycin (S)	35 (89.7)	4 (10.3)	0 (0.0)
Trimethoprim-sulfamethoxazole (SXT)	1 (2.6)	4 (10.3)	34 (87.1)
Tetracycline (TE)	0 (0.0)	4 (10.3)	35 (89.7)

*R, resistant; I, intermediate resistance; S, susceptibility.

### ERIC-PCR

**Figure 2:**
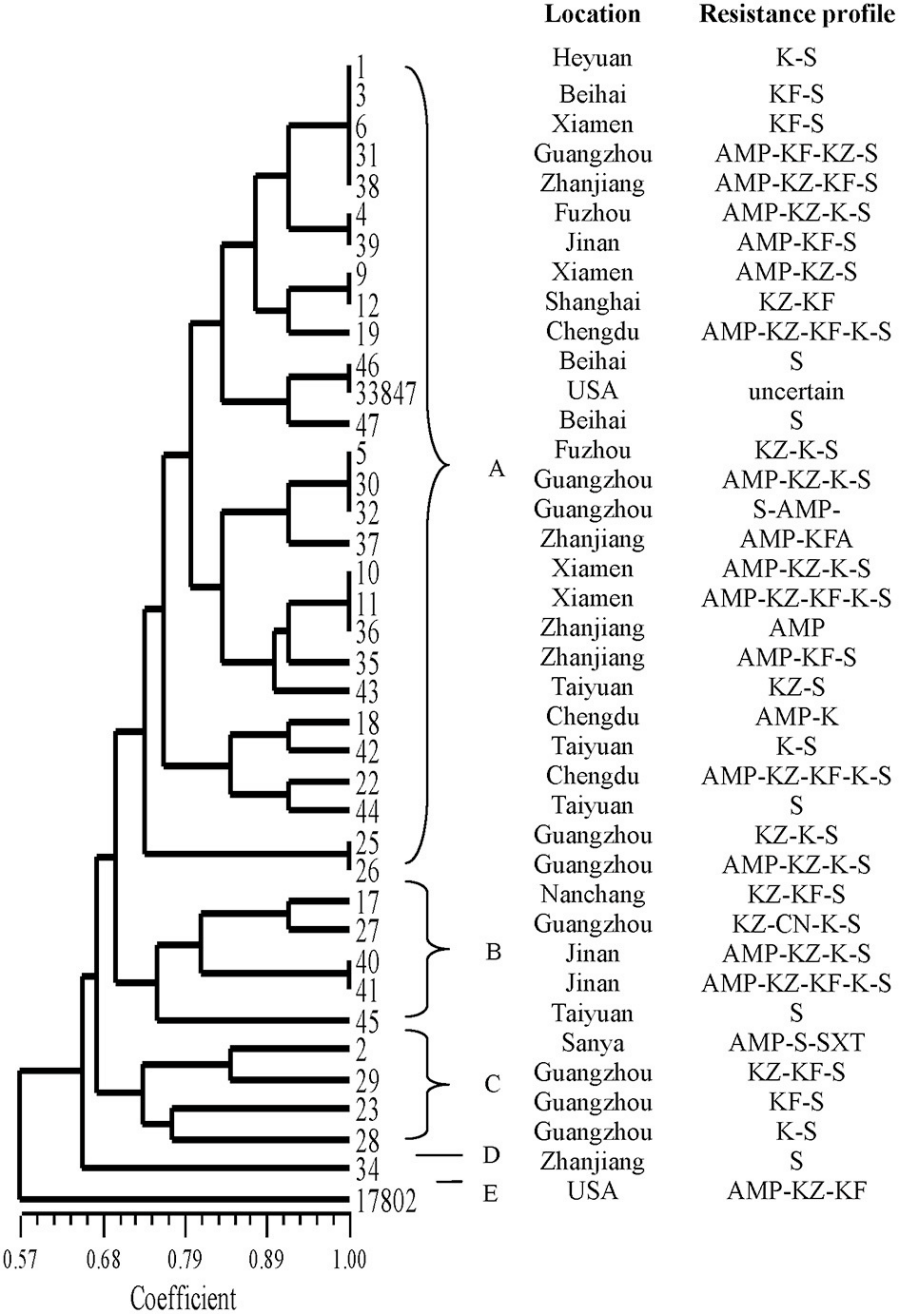
**ERIC-PCR DNA fingerprint analysis of ***V. parahaemolyticus*** isolates in RTE foods from China**.

The results of ERIC-PCR analysis of the 39 isolates are shown in Figure 2. ERIC-PCR resulted in 3–8 amplification bands, with sizes ranging from 130 to about 6000 bp. The ERIC image shows that bands with molecular sizes of 500, 1500, and 2500 bp were common (Figure S1). All the isolates were classified into five clusters (designated as A, B, C, D, and E). Most isolates were distributed on cluster A. Only one strain (no. 34) from Zhanjiang divided into cluster D. The standard strain ATCC 17802 was in cluster E alone. The other standard strain (ATCC 33847) was belonged on cluster A and on the same sub-cluster along with isolates nos. 46 and 47.

### MLST

**Figure 3:**
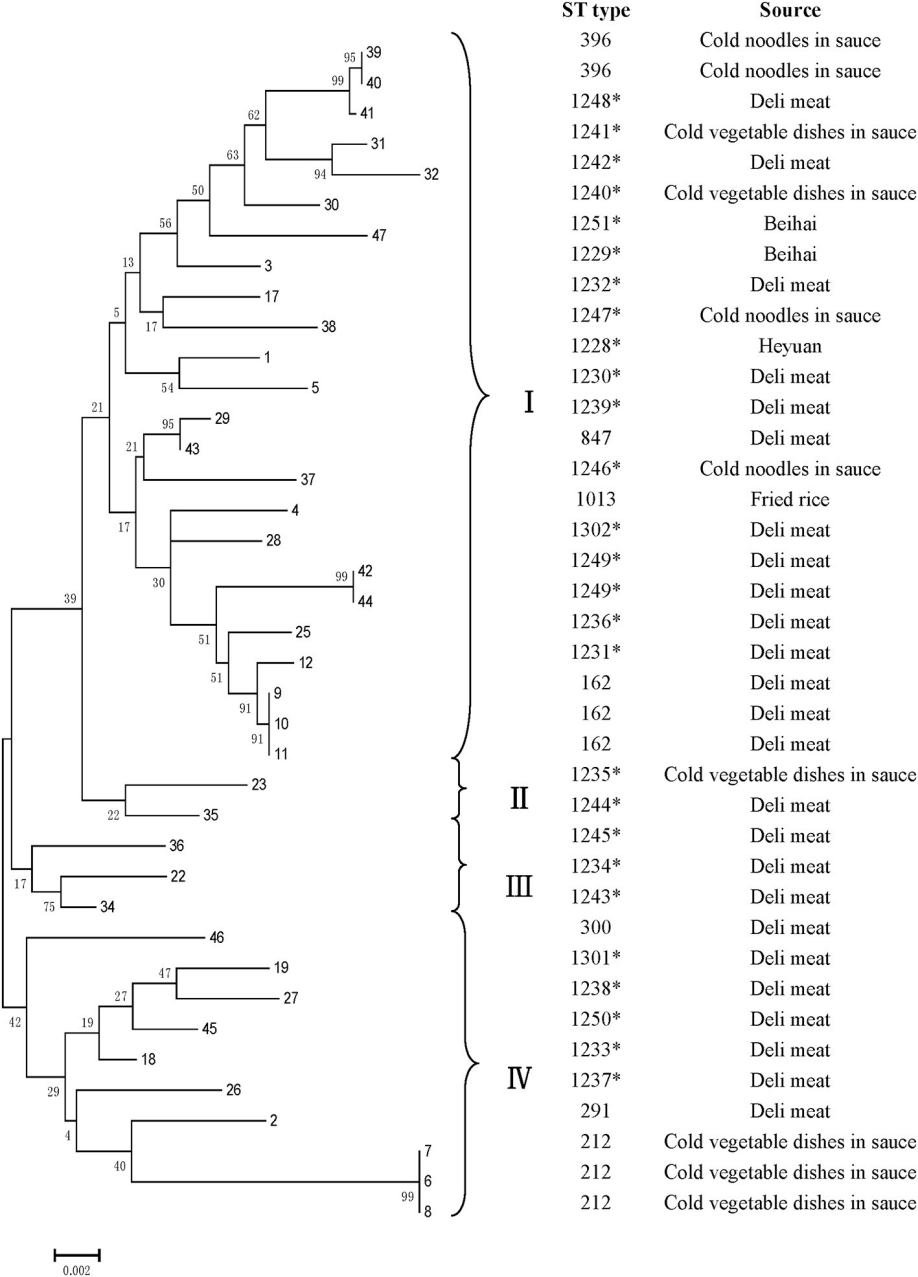
**MLST minimum evolution tree of the 39 V. ***parahaemolyticus*** RTE foods isolates**. The tree was built with Mega six software using concatenated sequences. ^*^ indicates the novel STs.

All *V. parahaemolyticus* isolates were analyzed by MLST using the sequences generated from internal fragments of seven HK genes. Numbers for alleles and sequence types (STs) were assigned according to the database created for *V. parahaemolyticus* on submitting the sequence results (Table 3). The potential novel loci would appear some mismatching base. Seven gene locus mismatching the ST types on database maybe the novel one. The administrator of the website was notified when we obtained novel loci and ST types; after confirmation, new numbers will be given. The numbers of alleles observed for each MLST locus in our study were distributed as follows: 22 (*dna E*), 22 (*gyr B*), 27 (*rec A*), 20 (*dtd S*), 18 (*pnt A*), 24 (*pyr C*), and 18 (*tna A*). Thirty-three STs were observed among the 39 isolates. There were ten novel loci: *dna E* 302, 303; *gyr B* 428; *rec A* 306, 307; d*td S* 371; *pnt A* 213; *pyr C* 339, 340; and *tna A* 238. Most of the isolates (26) were novel STs, namely 1228–1251, 1301, and 1302. Among all 3682 sites, the number of variable sites was 230; the total number of mutations was 245; the haplotype number was 33; and the haplotype diversity was 0.989. All loci showed ratios of synonymous and nonsynonymous substitutions (dN/dS) below 1. A minimum evolution tree was constructed using the concatenated sequences of each allele (Figure 3).

**Table 3:** **The MLST result of ***Vibrio parahaemolyticus*****.

**NO**.	**Name**	**Source**	**dnaE**	**gyrB**	**recA**	**dtdS**	**pntA**	**pyrC**	**tnaA**	**ST**
1	462tf	Deli meat	51	4	77	67	213^*^	8	24	1228^*^
2	VP002	Deli meat	60	170	133	145	2	130	26	291
3	642B3	Deli meat	42	88	113	242	18	99	23	1229^*^
4	VP004	Fried rice	60	284	4	53	43	63	23	1013
5	743tf	Deli meat	152	57	4	14	213^*^	54	14	1230^*^
6	VP006	Cold vegetable dishes in sauce	69	92	69	114	54	71	24	212
7	VP007	Cold vegetable dishes in sauce	69	92	69	114	54	71	24	212
8	VP008	Cold vegetable dishes in sauce	69	92	69	114	54	71	24	212
9	VP009	Deli meat	47	58	53	19	50	37	26	162
10	VP010	Deli meat	47	58	53	19	50	37	26	162
11	VP011	Deli meat	47	58	53	19	50	37	26	162
12	VP811	Deli meat	47	58	53	13	50	37	26	1231^*^
17	VP943	Deli meat	5	106	31	214	2	142	106	1232^*^
18	VP1029	Deli meat	31	106	135	19	26	62	54	1233^*^
19	VP1044B3	Deli meat	112	104	307^*^	13	23	339^*^	54	1301^*^
22	VP1409B3	Deli meat	302^*^	133	286	151	18	340^*^	37	1234^*^
23	VP1409	Cold vegetable dishes in sauce	11	75	64	151	124	7	50	1235^*^
25	VP1586A2	Deli meat	295	104	3	176	28	37	51	1236^*^
26	VP1588	Deli meat	26	428^*^	19	220	23	278	9	1237^*^
27	VP1588A1	Deli meat	5	106	307^*^	13	2	142	106	1238^*^
28	VP1588B3	Deli meat	44	260	31	67	26	200	99	1302^*^
29	VP1588C1	Deli meat	148	355	74	103	127	101	202	1239^*^
30	VP1589	Cold vegetable dishes in sauce	9	21	15	185	4	10	26	1240^*^
31	VP1609B1	Cold vegetable dishes in sauce	303^*^	25	246	185	31	252	73	1241^*^
32	VP1636	Deli meat	303^*^	25	306^*^	185	31	252	73	1242^*^
34	1787A3	Deli meat	132	16	286	371^*^	26	76	54	1243^*^
35	1787B2	Deli meat	11	106	192	19	71	73	17	1244^*^
36	1787C3	Deli meat	11	106	306^*^	220	71	73	17	1245^*^
37	1810	Cold noodles in sauce	36	131	44	67	102	5	37	1246^*^
38	1810C3	Cold noodles in sauce	126	25	123	103	103	7	26	1247^*^
39	VP039	Cold noodles in sauce	9	213	165	185	2	46	1	396
40	VP040	Cold noodles in sauce	9	213	165	185	2	46	1	396
41	2011A3	Deli meat	9	213	257	185	2	46	1	1248^*^
42	2138B2	Deli meat	44	58	257	371^*^	4	3	238^*^	1249^*^
43	VP043	Deli meat	148	355	74	19	127	101	202	847
44	VP044	Deli meat	44	58	257	371^*^	4	3	238^*^	1249^*^
45	2138C1	Deli meat	19	106	81	372^*^	31	5	33	1250^*^
46	VP046	Deli meat	14	30	67	46	27	11	13	300
47	2384A2	Deli meat	5	303	173	373^*^	152	318	23	1251^*^

“*” *The novel locis and STs*.

## Discussion

*V. parahaemolyticus* is a major seafood-borne gastroenteritis-causing bacterium that is frequently isolated from aquatic products (Letchumanan et al., 2015b). In our study, we analyzed 511 samples from Chinese RTE foods and isolated 39 *V. parahaemolyticus* strains. In our study, some samples only detected positive in the qualitative method. It may be related to the variation in culture concentration. Enrichment culture was streaked onto TCBS agar plates with undiluted liquid in qualitative method, while culture represent 1/10, 1/100, 1/1000 dilution were streaked onto TCBS agar plates in MPN method. For some samples were only positive by the MPN method, the reason maybe attribute to the incomplete homogeneous of the sample culture solution. When a loopful (10 μL) of bacterial suspension was streaked onto the TCBS agar plate could not certain to form colonies on the agar. A combination of qualitative and MPN methods avoid missing detection of *V. parahaemolyticus* effectively. Unlike *Listeria monocytogenes* (Shi et al., 2015) and *Salmonella* (Yang et al., 2016)*, V. parahaemolyticus* was not frequently detected on RTE foods. Previous study reported that no *V. parahaemolyticus* was positive in 145 samples of RTE food in Korea (Chung et al., 2010), similar result was found in Iran (Zarei et al., 2012). However, a report indicated that the prevalence of *V. parahaemolyticus* in RTE foods was an important cause of food poisoning in Shanghai, China (Tian et al., 2008). As we know, RTE foods do not need further processing before consumption. Thus, identification of contamination in RTE foods is critical for assuring food safety. Our research can provide insights into food safety in RTE foods.

TDH and TRH are considered major virulence factors in *V. parahaemolyticus* (Ceccarelli et al., 2013; Letchumanan et al., 2014; Raghunath, 2014). Moreover, the presence of *tdh*- and/or *trh*-positive *V. parahaemolyticus* strains represents a major public health risk. In our study, strains identified in Chinese RTE foods strains were negative for both *trh* and *tdh* genes. These results are consistent with the findings of a previous study reported in India (Raghunath et al., 2008), but this was contradictory to most findings from other previous studies (Zhao et al., 2011; Letchumanan et al., 2015a). The overall mechanism of *V. parahaemolyticus* pathogenesis remains unclear (Ceccarelli et al., 2013); although TDH and TRH have been shown to be correlated with pathogenic strains, they do not fully explain the pathogenicity of *V. parahaemolyticus* (Lynch et al., 2005). Several studies have reported the presence of clinical strains without *tdh* and *trh* (Shirai et al., 1990). Thus, even in the absence of *tdh* or *trh, V. parahaemolyticus* still remains pathogenic, and some environmental isolates lacking *tdh* and/or *trh* can produce putative virulence factors. For example, some oyster isolates will contain T3SS1 genes without *tdh* and/or *trh* (Mahoney et al., 2010; Jones et al., 2012).

In 1996, pandemic O3:K6 serovars were shown to be responsible for *V. parahaemolyticus* outbreaks. The serotype O3:K6 *V. parahaemolyticus* emerged from India and spread throughout the world, including to China and the USA (Okuda et al., 1997; Honda et al., 2008; Tan et al., 2010). Our study indicated that serovar O2 was the predominant serotype among the strains isolated from RTE foods. These findings are in contrast with the results of a previous study, in which the O3 serotype was identified as the predominant serotype in China (Zhang et al., 2006; Zhao et al., 2011). O3:K6, O1:Kut, O4:K8, and O2:K3 were also the dominant serovars identified in outbreaks of *V. parahaemolyticus* in China (Zhang et al., 2013; Ma et al., 2014). The O3 isolate was the same as the most frequent serotype among our clinical isolates (source: Shenzhen Centres for Disease Control). We also showed the presence of other O-type serovars of *V. parahaemolyticus* from RTE foods; the results demonstrated the diverse distributions in different RTE food types and locations in China. The changes in pandemic serogroups of *V. parahaemolyticus* have been reported to occur over time, an increasing number of nonpandemic serogroups have been shown to carry pandemic marker genes (Matsumoto et al., 2000). Therefore, these O2 strains from Chinese RTE foods may have the potential for pathogenesis in humans.

With the steady expansion of the Asian aquaculture industry, in order to increase production, aquaculture farmers are using different antibiotics to prevent (prophylactic use) and treat (therapeutic use) pathogenic bacterial infections in aquatic products (Cabello et al., 2013; Huang et al., 2015; Tan et al., 2016). Furthermore, the continuous and extensive use of antibiotics in humans, has led to the emergence of antimicrobial-resistant *V. parahaemolyticus* strains worldwide (Sani et al., 2013; Yano et al., 2014). In our study, the highest resistance rate was observed for streptomycin. Similarly, previous study have demonstrated the occurrence of streptomycin- and ampicillin-resistant *V. parahaemolyticus* isolates (Pazhani et al., 2014). We also found a small number of isolates showing resistance to gentamicin and trimethoprim-sulfamethoxazole, which are first-line drugs used in clinic treatment. Moreover, we found that most of the strains (21/39 isolates) were multidrug resistant. As RTE foods are eaten without cooking, the presence of these strains will increase the health risks of consuming such foods in humans. Thus, it may be important to evaluate variations in antimicrobial susceptibility profiles in *V. parahaemolyticus* strains.

Molecular subtyping is widely used for the analysis of genetic diversity. ERIC-PCR provides discriminatory values and can be used for rapid *V. parahaemolyticus* typing (Khan et al., 2002). Compared to *V. parahaemolyticu*s isolates from the 1997 Canadian outbreak using three method, they found that ERIC-PCR and ribotyping were the most informative typing methods (Marshall et al., 1999). Using this approach, the isolates were classified into five clusters at 0.65 similarity. Most of the strains were on cluster A; which showed they were may be genetically related. The reference strain ATCC 17804 harbors *trh* grouped into a single cluster E, exhibiting differences with other strains. Strain (no. 34), from Zhanjiang, was grouped into cluster D alone and was genetically diverse from other isolates. Clustering based on ERIC-PCR did not coincide with the isolation sources or patterns of antibiotic resistance. The result was similar to other studies, showing the high genetic diversity in *V. parahaemolyticus* strains. MLST is a good method for typing owing to its reproducibility, as shown in the sequencing of seven housekeeping genes; this method has been widely used for analysis of the *V. parahaemolyticus* sequence (Lüdeke et al., 2015). In our study, all the isolates could be grouped into four main clusters (I, II, III, and IV). ST 291, ST 396, and ST 300 were identified in a public database as environmental isolates from China; ST1013 was separated from environmental samples from USA. Thus, some *V. parahaemolyticus* isolates from RTE foods were similar to environment strains. Additionally, one ST162 strain was reported as a clinical isolate from the USA. With the identification of 10 novel loci and 22 novel STs, this study substantially contributed to the diversity in the MLST database. As most strains identified in RTE foods *V. parahaemolyticus* stains were novel STs, these results suggested that *V. parahaemolyticus* strains found in RTE foods may be distinct from other stains. In our study, both ERIC-PCR and MLST confirmed the genetic diversity within strains.

In summary, this is the first comprehensive study that described the prevalence, serotype, virulence, antibiotic resistance phenotype, and molecular subtyping of *V. parahaemolyticus* from RTE foods in China. This study showed that none of the isolates possessed *tdh* and *trh*, and serotype O2 was found to be prevalent. The antimicrobial-resistance patterns revealed that the streptomycin-resistant were widespread (89.7%) and the isolates resistance to some clinical antibiotics such as cephalothin, gentamicin. ERIC-PCR and MLST typing showed genetic diversity. The novel loci and STs indicated genetically diverse on RTE foods isolates. As RTE foods are common and popular food choices in China, therefore the continuous monitoring of food-borne pathogens such as *V. parahaemolyticus* are vital to ensure the safety of these food products.

## Author contributions

TX, XX are the common first authors, finsh the article experiment and write the article together. QW (Corresponding Author) give the idea and experiments support. JZ, JC help to finish the experiment on article.

### Conflict of interest statement

The authors declare that the research was conducted in the absence of any commercial or financial relationships that could be construed as a potential conflict of interest.
