# Supplementary material for: Prevalence, Molecular Characterization, and Antibiotic Susceptibility of Vibrio parahaemolyticus from Ready-to-Eat Foods in China
Source: Front Microbiol. 2016 Apr 21;7:549. doi: 10.3389/fmicb.2016.00549 (PMC4839030; doi:10.3389/fmicb.2016.00549)
Supplement: Supplementary file 1 [file DataSheet1.doc]

**Supplementary Materials**

**Tables**

**Table S1 Seven oligonucleotide primer sequences of multilocus sequence typing**

| Gene | | Primer | Length (bp) |
| --- | --- | --- | --- |
| *rec A* | *rec A*-1F | GAAACCATTTCAACGGGTTC | 773 |
|  | *rec A*-1R | CCATTGTAGCTGTACCAAGCACCC |  |
| *gyr B* | *gyr B*-1F | GAAGGBGGTATTCAAGC | 629 |
|  | *gyr B*-1R | GAGTCACCCTCCACWATGTA |  |
| *dna E* | *dna E*-1F | CGRATMACCGCTTTCGCCG | 596 |
|  | *dna E*-1R | GAKATGTGTGAGCTGTTTGC |  |
| *dtd S* | *dtd S*-1F | TGGCCATAACGACATTCTGA | 497 |
|  | *dtd S*-1R | GAGCACCAACGTGTTTAGC |  |
| *pnt A* | *pnt A*-1F | ACGGCTACGCAAAAGAAATG | 470 |
|  | *pnt A*-1R | TTGAGGCTGAGCCGATACTT |  |
| *pyr C* | *pyr C*-1F | AGCAACCGGTAAAATTGTCG | 533 |
|  | *pyr C*-1R | CAGTGTAAGAACCGGCACAA |  |
| *tna A* | *tna A*-1F | TGTACGAAATTGCCACCAAA | 463 |
|  | *tna A*-1R | AATATTTTCGCCGCATCAAC |  |

**Table S2. Results of serotyping, antimicrobial resistance, and ERIC-typing of *Vibrio parahaemolyticus*** isolates in this study

| NO. | Source | Location | Resistance profile^b^ | MAR  index | ERIC | Serotyping^a^ | ST |
| --- | --- | --- | --- | --- | --- | --- | --- |
| 1 | Deli meat | Heyuan | K-S | 0.17 | A | O1 | 1228 |
| 2 | Deli meat | Sanya | AMP-S-SXT | 0.25 | C | O2 | 291 |
| 3 | Deli meat | Beihai | KF-S | 0.17 | A | O12 | 1229 |
| 4 | Fried rice | Fuzhou | AMP-KZ-K-S | 0.33 | A | O12 | 1013 |
| 5 | Deli meat | Fuzhou | KZ-K-S | 0.25 | A | O12 | 1230 |
| 6 | Cold vegetable dishes in sauce | Xiamen | KF-S | 0.17 | A | O1**2** | 212 |
| 7 | Cold vegetable dishes in sauce | Xiamen | S | 0.08 | uncertain | O1**2** | 212 |
| 8 | Cold vegetable dishes in sauce | Xiamen | AMP-KZ-KF-S | 0.33 | uncertain | O1**2** | 212 |
| 9 | Deli meat | Xiamen | AMP-KZ-S | 0.25 | A | O4 | 162 |
| 10 | Deli meat | Xiamen | AMP-KZ-K-S | 0.33 | A | O4 | 162 |
| 11 | Deli meat | Xiamen | AMP-KZ-KF-K-S | 0.42 | A | O4 | 162 |
| 12 | Deli meat | Shanghai | KZ-KF | 0.17 | A | O4 | 1231 |
| 17 | Deli meat | Nanchang | KZ-KF-S | 0.25 | B | O2 | 1232 |
| 18 | Deli meat | Chengdu | AMP-K | 0.17 | A | O2 | 1233 |
| 19 | Deli meat | Chengdu | AMP-KZ-KF-K-S | 0.42 | A | O4 | 1301 |
| 22 | Deli meat | Chengdu | AMP-KZ-KF-K-S | 0.42 | A | O2 | 1234 |
| 23 | Cold vegetable dishes in sauce | Guangzhou | KF-S | 0.17 | C | O6 | 1235 |
| 25 | Deli meat | Guangzhou | KZ-K-S | 0.25 | A | O4 | 1236 |
| 26 | Deli meat | Guangzhou | AMP-KZ-K-S | 0.33 | A | O4 | 1237 |
| 27 | Deli meat | Guangzhou | KZ-CN-K-S | 0.33 | B | O7 | 1238 |
| 28 | Deli meat | Guangzhou | K-S | 0.17 | C | O2 | 1302 |
| 29 | Deli meat | Guangzhou | KZ-KF-S | 0.25 | C | O2 | 1239 |
| 30 | Cold vegetable dishes in sauce | Guangzhou | AMP-KZ-K-S | 0.33 | A | O5 | 1240 |
| 31 | Cold vegetable dishes in sauce | Guangzhou | S-KF-AMP-KZ | 0.33 | A | O2 | 1241 |
| 32 | Deli meat | Guangzhou | S-AMP- | 0.33 | A | O12 | 1242 |
| 34 | Deli meat | Zhanjiang | S | 0.17 | A | O2 | 1243 |
| 35 | Deli meat | Zhanjiang | S-KFAMP | 0.08 | D | O2 | 1244 |
| 36 | Deli meat | Zhanjiang | AMP | 0.25 | A | O5 | 1245 |
| 37 | Cold vegetable dishes in sauce | Zhanjiang | AMP-KF | 0.08 | A | O5 | 1246 |
| 38 | Cold vegetable dishes in sauce | Zhanjiang | AMP-KZ-KF-S | 0.17 | A | O1 | 1247 |
| 39 | Cold vegetable dishes in sauce | Jinan | AMP-KF-S | 0.33 | A | O8 | 396 |
| 40 | Cold vegetable dishes in sauce | Jinan | AMP-KZ-K-S | 0.25 | A | O8 | 396 |
| 41 | Deli meat | Jinan | AMP-KZ-KF-K-S | 0.33 | B | O**1** | 1248 |
| 42 | Deli meat | Taiyuan | K-S | 0.42 | B | O2 | 1249 |
| 43 | Deli meat | Taiyuan | KZ-S | 0.17 | A | O2 | 847 |
| 44 | Deli meat | Taiyuan | S | 0.17 | A | O2 | 1249 |
| 45 | Deli meat | Taiyuan | S | 0.08 | A | O2 | 1250 |
| 46 | Deli meat | Beihai | S | 0.08 | B | O4 | 300 |
| 47 | Deli meat | Beihai | S | 0.08 | A | O3 | 1251 |
| 17802 | ATCC | USA | AMP-KZ-KF | 0.25 | E | O1 |  |
| 33847 | ATCC | USA | uncertain |  | A | O4 |  |

^a^O3 or O13

^b^For antimicrobial abbreviation, Azitromycin (AZM), Cefazolin (KZ), Cephalothin (KF), Chloramphenicol (C), Ciprofloxacin (CIP), Gentamicin (CN), Kanamycin (K), Nalidixic acid (NA), Streptomycin (S), Trimethoprim-sulfamethoxazole (SXT), Tetracycline (TE); /, no resistance; nd: not determined

NO. 6, 7, and 8; NO. 26, 27, 28, and 29; NO. 35 and 36; NO. 37 and 38; NO. 39 and 40; NO. 43 and 44 isolated from same sample, respectively.


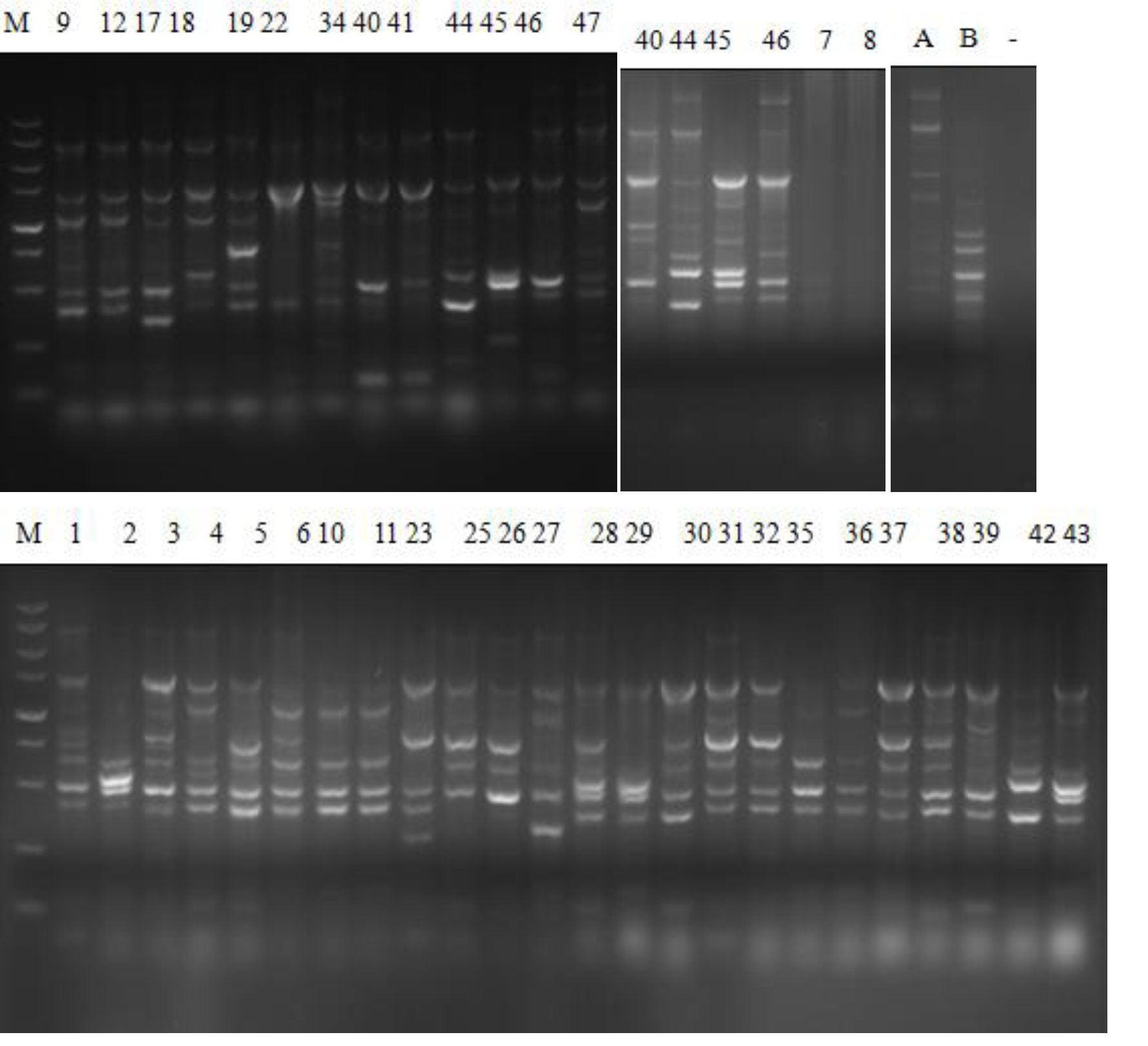


**Fig.S1. ERIC-typing of *Vibrio parahaemolyticus* isolates in this study**

“M” 5000bp marker.

A ”stand for 33847; “B” stand for 17802; “-” stand for negative control.


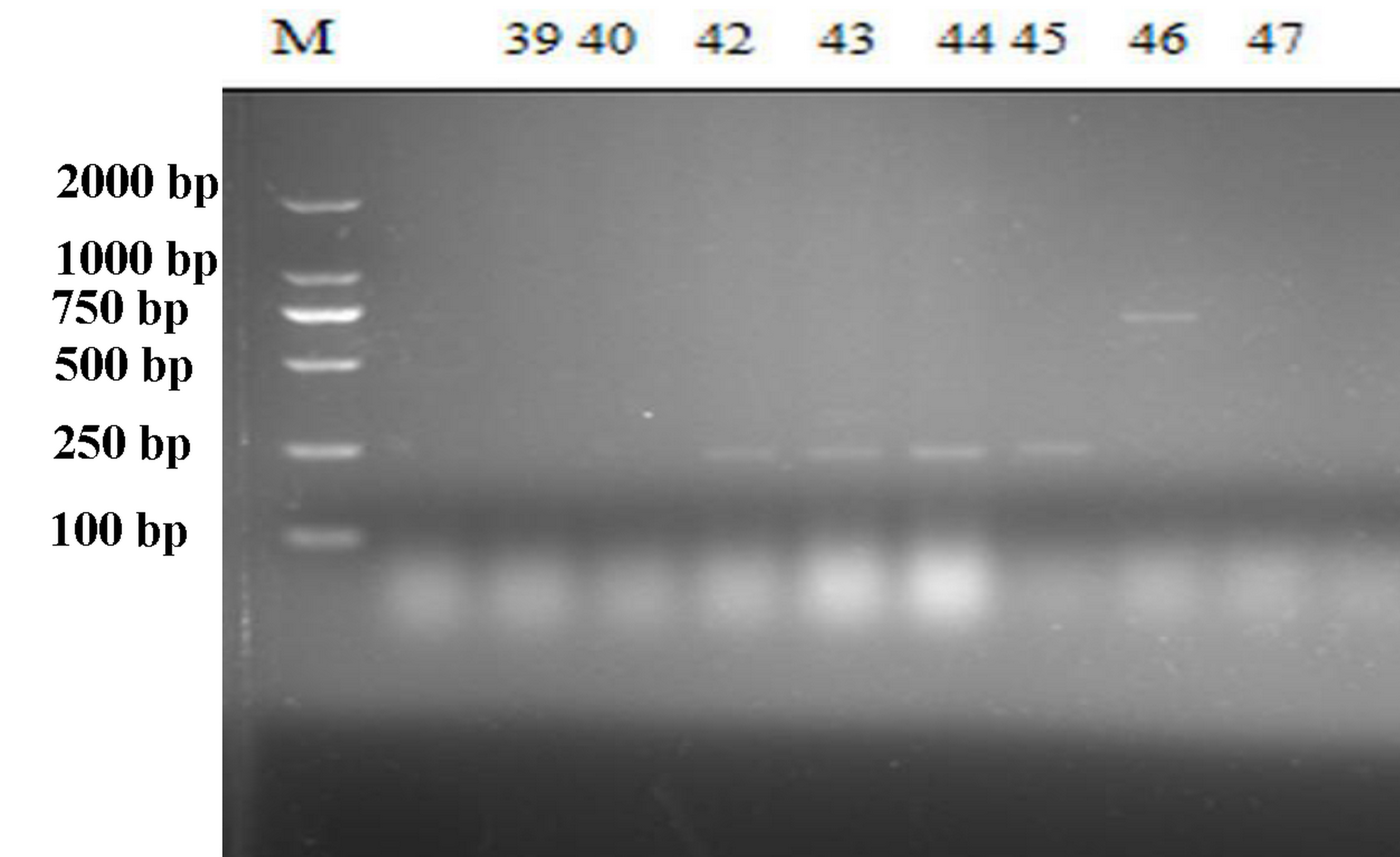


**Fig.S2. Part of *Vibrio parahaemolyticus* isolates serotyping PCR gelsin this study (Group 1).**

“M” 2000bp marker.
